# The synergistic effect of IFN-α and IFN-γ against HSV-2 replication in Vero cells is not interfered by the plant antiviral 1-cinnamoyl-3, 11-dihydroxymeliacarpin

**DOI:** 10.1186/1743-422X-3-45

**Published:** 2006-06-13

**Authors:** Erina Petrera, Celia E Coto

**Affiliations:** 1 Laboratory of Virology, Department of Biochemistry, School of Science, University of Buenos Aires, Buenos Aires, Argentina

## Abstract

**Background:**

Recent studies have shown that gamma interferon (IFN-γ) synergizes with IFN-α/β to inhibit herpes simplex virus type 1 (HSV-1) replication *in vitro*. Since IFN response represents an early host defense event against viral infection and the fact that treatment with meliacine, a plant antiviral, ameliorate the severity of the herpetic infection in female mice infected intravaginally with HSV-2, we wanted to investigate whether the administration of meliacine to HSV-2 infected mice could altered the homoestasis of IFNs host response. For this purpose we studied the effect of the compound 1-cinnamoyl-3,11-dihydroxymeliacarpin (CDM), which is the responsible for meliacine antiviral action, on the HSV-2 inhibition exerted by IFN α, IFN-γ or their combination.

**Results:**

We have found that like HSV-1, IFN-γ synergizes with IFN-α to inhibit HSV-2 replication in Vero cells. While treatment with IFN-α or IFN-γ alone has weak antiviral action, HSV-2 plaque formation, viral replication and the onset of viral CPE in Vero cells are synergistically inhibited by interferon combination. In addition, CDM treatment contributes to protect cells from virus cytopathic effect and causes a strong inhibition of HSV-2 titer. Moreover, the presence of CDM for 2 h before IFN induction, during the 16 h induction period, only for 24 h after infection or during the complete IFN treatment period, reduces virus yields in an additive way without affecting IFN antiviral action.

**Conclusion:**

The results reported here indicated that the presence of CDM did not alter the antiviral activity of IFN-α, IFN-γ or the synergism exerted by their combination. As a result we can envision that the administration of CDM *in vivo *could not affect the biological activity of IFNs, which are so important mediators of the innate resistance to HSV-2 infection.

## Background

Herpes simplex virus type 2 (HSV-2) is a sexually transmitted pathogen that infects both the oral and genital mucosa of humans and is a significant cause of morbidity worldwide. A mouse vaginal model of HSV-2 infection has been developed by several investigators [1-4]. Although the degree of pathogenicity of the virus for mice is dependent on the virus strain used, in general, experimental infection by vaginal route (i.v) results in neurological disease, which is preceded by easily recognizable symptoms due to inflammation followed by rear leg paralysis and death. This mouse model provides a useful tool to test the effect of antivirals against HSV-2 infection.

Many studies have been performed in our laboratory with an antiviral compound isolated from the leaves of *Melia azedarach *L. named meliacine (MA). We have shown that meliacine strongly inhibited the replication of HSV-1 and HSV-2 in Vero cells [5] and exhibits a synergistic antiviral activity when combined with acyclovir [6]. Studies performed by Alché *et al *suggested that MA exerts the antiviral action on both synthesis of viral DNA and maturation and progress of HSV-1 on Vero cells [7]. *In vivo *studies have shown that meliacine prevents the development of HSV-1 stromal keratitis in mice [8,9]. Likewise, the severity of the herpetic infection in female mice infected intravaginally with HSV-2 was also ameliorated by MA treatment [10]. On the other hand, besides its broad effect of antiviral action, meliacine acts as an immunomodulator in vitro agent inhibiting the phagocytosis of opsonized sheep erythrocytes and impairing the proliferation of spleen and lymph node T cells [11,12]. Moreover, meliacine is a weak inducer of tumor necrosis factor alpha (TNF-α) in murine macrophage cultures and causes a synergistic effect on the production of TNF-α induced by LPS [13]. Vaginal washes of female mice infected i.v. with HSV-2 and treated with meliacine contained an increased amount of TNF-α in comparison with infected non-treated animals [10].

IFN response represents an early host defense event, one that occurs prior to the onset of the immune response. In this context, macrophages play a central role in resistance of mice to primary infection with HSV-2, mainly, as a source of antiviral cytokines, TNF-α, IFN α/β and IL-12, which are produced rapidly after infection [14]. IFN-γ, a strong activator of macrophages [15-17] is produced both in the early stages of infection by natural killer cells and at later stages by activated T cells [18]. The innate immune response to viral infection depends on the integrity of this network of cytokines, which is tightly regulated [19]. This *in vivo *situation led us to query whether the administration of meliacine to HSV-2 infected mice could altered the homoestasis of IFNs host response either affecting the antiviral activity of IFN α/β or IFN-γ, or their synergizing interaction [20,21]. To answer that question we conducted experiments following an indirect approach based on the observation that IFN-γ synergizes with IFN α/β to inhibit HSV-1 replication in Vero cells [20]. To that end, Vero cells infected with HSV-2 were treated with IFN-α, IFN-γ or a combination of both in the presence or absence of meliacine under different experimental conditions.

To perform these experiments instead of meliacine, we worked with the compound 1-cinnamoyl-3,11-dihydroxymeliacarpin (CDM) which is the molecule responsible for the broad spectrum of meliacine antiviral action [22]. In summary, here we analyzed: *i) *the susceptibility of HSV-2 to IFN-α, IFN-γ or the combination of both in Vero cells since no published data is available with this herpesvirus; *ii) *the effect of CDM on interferons action.

## Results

### Antiviral effect of IFN-α, IFN-γ and IFNs combination on HSV-2 plaque formation

Since there is no published data on the effect of IFNs on HSV-2 infection in Vero cells the capacity of human IFN-α and/or IFN-γ to inhibit the replication of HSV-2 strains MS and G was initially performed in a plaque reduction assay. The concentration of IFN-α and IFN-γ used in the present experiment were those previously tested against HSV-1 (KOS strain) [20]. Vero cells were pretreated for 16 hours with 100 IU/ml of IFNs separately or in combination and infected with HSV-2 (MS or G strain) at the MOI of 1 PFU per cell. HSV-1 (KOS and F strains) were also tested using the same MOI and served as controls. The efficiency of HSV-1 strains KOS and F plaque formation was quite modestly reduced by the presence of IFN-α or IFN-γ alone. Whereas, the combination of IFN-α and IFN-γ acted synergistically as previously reported for HSV-1 [20,23].

Simultaneous treatment of Vero cells with both IFN-α and IFN-γ reduced HSV-2 plaque formation 3.8 fold for MS strain and 8.6 fold for G strain in comparison with the effect of each IFN alone (Table 1). Likewise HSV-1, the level of inhibition achieved with IFN-α and IFN-γ combination treatment was not a consequence of doubling the amount of IFN per culture. As seen in Table 1, increasing the concentration of each IFN to 200 IU/ml did not augment the inhibitory effect.

**Table 1:** Effect of IFN-α and IFN-γ on HSV-2 and HSV-1 plaque formation on Vero cells.

	Treatment (IU/ml)^a^	Mean no. of plaques^b ^± SEM	Fold reduction^c^
HSV-2 MS	Vehicle	137 ± 8.3	-
	IFN-α (100)	82.5 ± 6.4	1.7
	IFN-γ (100)	86 ± 6.6	1.6
	IFN-α (100)+IFN-γ (100)	36.5 ± 4.3	**3.8**
	IFN-α (200)	68 ± 5.8	2
	IFN-γ (200)	72 ± 6	1.9
HSV-2 G	Vehicle	98.5 ± 7	-
	IFN-α (100)	81 ± 6.4	1.2
	IFN-γ (100)	98 ± 7	1
	IFN-α (100) + IFN-γ (100)	11.5 ± 2.4	**8.6**
	IFN-α (200)	52 ± 5.1	1.9
	IFN-γ (200)	35 ± 4.2	2.8
HSV-1 KOS	Vehicle	133 ± 8.2	-
	IFN-α (100)	68.5 ± 5.9	1.9
	IFN-γ (100)	74.5 ± 6.1	1.8
	IFN-α (100) + IFN-γ (100)	4 ± 1.4	**33**
	IFN-α (200)	54.5 ± 5.2	2.4
	IFN-γ (200)	84.5 ± 6.5	1.6
HSV-1 F	Vehicle	100 ± 7.1	-
	IFN-α (100)	82.5 ± 6.4	1.2
	IFN-γ (100)	84.5 ± 6.5	1.2
	IFN-α (100) + IFN-γ (100)	19 ± 3.1	**5.3**
	IFN-α (200)	51.5 ± 5.1	1.9
	IFN-γ (200)	53 ± 5.1	1.9

^a^Vero cells were treated continuously with IFN-α, IFN-γ or combinations of these cytokines from 16 h before infection until the end of the experiment.

^b ^Average number of plaques per well of Vero cells inoculated with 200 PFU of HSV-1 KOS and F and HSV-2 MS and G. Values represent (mean ± SEM) from three independent experiments.

^c ^Fold-reduction in each group was calculated as follows: number of plaques in vehicle/number of plaques in treatments. Values represent mean from three independent experiments. P < 0.05, as determined by one-way ANOVA and Turkey's post hoc *t *test comparison of this treatment to vehicle.

These results indicate that HSV-2 replication in Vero cells, as other members of the herpes virus family like HSV-1 [20], VZV [24] and CMV [25] shows an increased susceptibility to IFN combination with respect to each IFN alone.

### Effect of IFN-α and IFN-γ on HSV-2 replication

**Figure 1:**
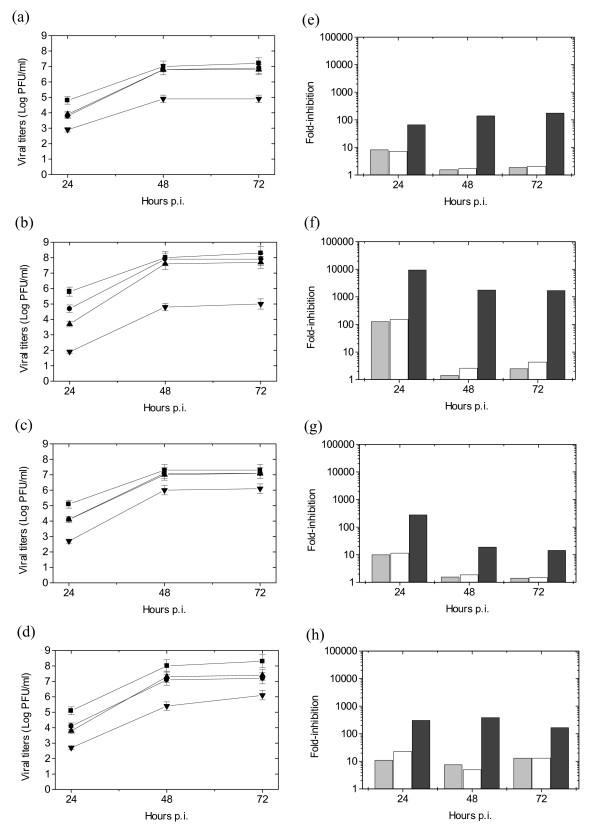
Effect of IFN-α and IFN-γ on HSV replication. Vero cells were treated with (■) vehicle or 100 IU/ml each of (●) IFN-α, (▲) IFN-γ or (▼) IFN-α and IFN-γ 16 h before infection with HSV-2 strain (a, e) MS, HSV-2 strain (b, f) G, HSV-1 strain (c, g) KOS or HSV-1 strain (d, h) F at a MOI of 1 PFU per cell. Supernatants were harvested on the indicated days p.i. and viral titers were determined by plaque assay as described in Material and Methods. (e-h) Average fold inhibition in viral replication observed in cells treated 100 IU/ml each of () IFN-α, (□) IFN-γ or (■) IFN-α and IFN-γ was calculated as (average viral titers in vehicle-treated/average viral titers in IFN-treated). One-way ANOVA followed by Tukey's post hoc *t *test confirmed that the differences were significant (p < 0.001).

To further characterize the inhibitory effect of IFN-α and IFN-γ treatment on HSV-2 replication, three days viral growth assay were performed. Vero cells were pretreated for 16 h with 100 IU/ml of IFNs separately or in combination. Then, cells were infected with HSV-2 (MS or G strain) at the MOI of 1 PFU per cell and culture supernatants were harvested at 24, 48 and 72 h p.i. and titered for infectious virus. For control purpose HSV-1 strains KOS and F similarly treated were included. In accordance with the results presented in Table 1, we observed a greater inhibitory effect on HSV-2 replication when Vero cultures were treated with IFNs combination than IFN-α or IFN-γ alone, despite that each interferon showed a greater antiviral activity than in plaque reduction assay (Figure 1). In cultures treated with 100 IU/ml of IFN-α or IFN-γ, MS and G replication was 8-fold and 100-fold reduced respectively (p < 0.001) at 24 h p.i. (Figure 1a and 1b). At 48 and 72 h p.i. viral titers in IFN-α or IFN-γ-treated cultures approached levels of those detected in vehicle-treated groups. However, relative to vehicle control cultures, viral titers recovered at 48 and 72 h from cultures treated with IFN-α or IFN-γ were reduced by 2-fold in MS-infected cultures and 2-and 3-fold in G infected cultures respectively (Figure 1e and 1f). In HSV-2 infected cultures treated with combination of IFN-α and IFN-γ the inhibitory effect was different between strains. Titers of HSV-2 MS were reduced 100-fold approximately relative to vehicle treated Vero cells at all time point tested. In the case of HSV-2 G, IFN combination virus titers were reduced 10.000-fold in comparison to control cultures at 24 h p.i. At 48 and 72 h p.i. the antiviral activity decreased, however virus replication was still 2000-fold inhibited. These results indicate that the combination of IFN-α and IFN-γ synergizes the antiviral effect against HSV-2 in a similar mode to previously reported for HSV-1 (Figure 1c, 1d, 1g and 1h) [23,25].

### Treatment with IFN combination protects HSV-2 infected cells from viral cytopathic effect (CPE)

**Figure 2:**
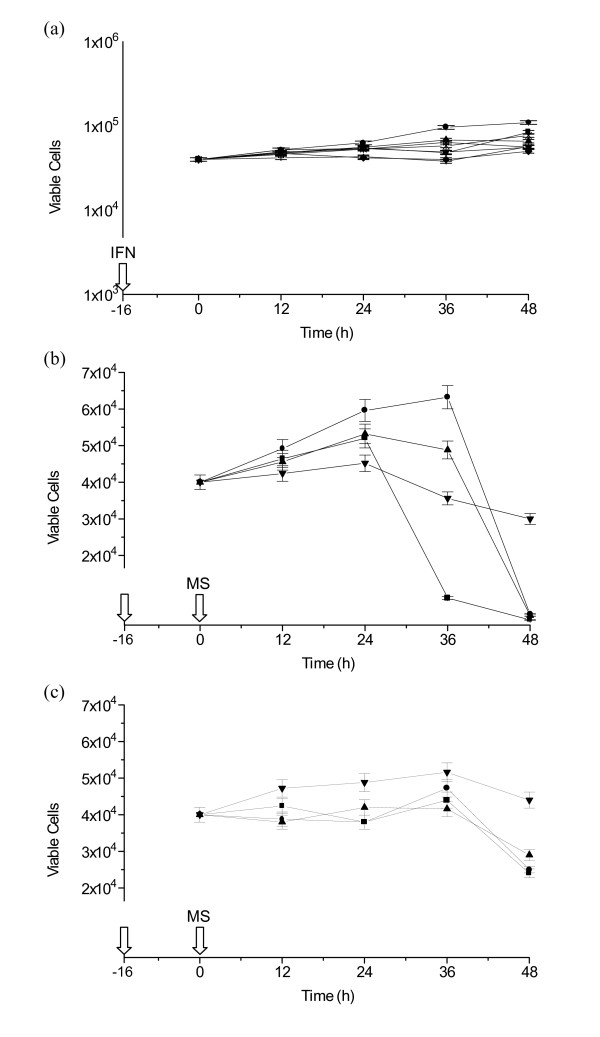
Treatment with IFN protects HSV-2 infected cells from viral cytopathic effect. (a) Uninfected cells per culture that remained viable at different times after treatment with (■) vehicle, (●) 100 IU/ml IFN-α, (▲) 100 IU/ml IFN-γ, () 100 IU/ml each of IFN-α and IFN-γ, (▼) CDM, (◆) CDM + IFN-α, (+) CDM + IFN-γ and () CDM + IFN-α and IFN-γ. (b) Number of MS-infected cells that remained viable at times after inoculation in the presence of (■) vehicle, (●) 100 IU/ml IFN-α, (▲) 100 IU/ml IFN-γ or (▼) 100 IU/ml each of IFN-α and IFN-γ. (c) Number of MS-infected cells that remained viable at times after inoculation in the presence of (■) CDM, (●) CDM + 100 IU/ml IFN-α, (▲) CDM + 100 IU/ml IFN-γ or (▼) CDM + 100 IU/ml each of IFN-α and IFN-γ.). One-way ANOVA followed by Tukey's post hoc *t *test confirmed that the differences were significant (p < 0.001).

Due to the long time that IFNs were in contact with cells in the experiments just described we performed new ones in order to discard any cytotoxicity due to the cytokines that could affect the results observed. For that purpose cultures of Vero cells were treated for 16 h with IFN-α, IFN-γ or the combination of both. After that time, monolayers were infected with 1 PFU per cell of HSV-2 MS strain and fresh medium containing or not IFN was added after 1 h virus adsorption and remained up to the end of the experiment. Cell morphology was observed by light microscope and the number of viable cells at 0, 12, 24, 36 and 48 h p.i. was determined by MTT colorimetric assay. In comparison with vehicle treated uninfected cells, IFN treatment did not affect cell morphology or proliferation. (Figure 2a). Infection with HSV-2 MS strain destroyed 87.5% of vehicle-treated cells by 48 h p.i. (Figure 2b). Treatment with IFN-α or IFN-γ delayed the onset of CPE in MS-infected cultures and increased the fraction of viable cells recovered between 24 and 36 h p.i. (Figure 2b). However, at 48 h p.i. the cultures appeared like vehicle treated cells. By contrast, a combination of IFN-α and IFN-γ provided the greatest protection, 75% of MS-infected cells remained viable at 48 h p.i. (Figure 2b).

The results of these experiments supported the previous findings (Table 1 and Figure 1) that combination of IFN-α and IFN-γ inhibited HSV-2 replication in Vero cells in a synergistic manner.

### Effect of IFN-α, IFN-γ and IFN combination on HSV-2 replication in the presence of CDM

In order to investigate whether or not CDM could interact with cytokines antiviral effect in Vero cells we performed several experiments. First, we tested the effect of CDM on cell CPE protection provided by IFN-α, IFN-γ or the combination of both. For this purpose an experiment similar to that described in Figure 2b was performed but in the presence of CDM (50 μg/ml). The number of surviving cells was determined at 12, 24, 36 and 48 h p.i. (Figure 2c). A comparison of Figure 2b and 2c shows that in the presence of CDM alone Vero cells were highly protected from HSV-2 cytopathicity, since 60% of infected cells remained viable. When CDM was combined with IFN-α or IFN-γ, the number of protected cells increased with respect to the effect of each interferon alone suggesting that the protection observed is due to CDM effect. Interestingly, when cells were treated with CDM plus IFN combination there seems to be an additive antiviral effect, a phenomenon that was confirmed by next experiments.

**Figure 3:**
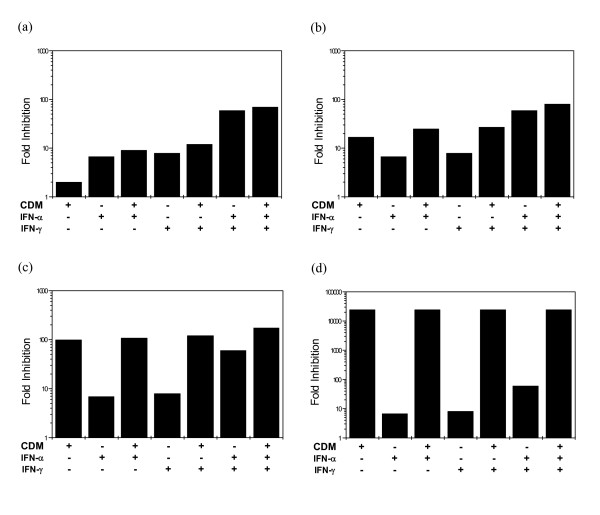
Effect of CDM on the antiviral action exerted by IFNs. Vero cells were treated with 100 IU/ml of IFN-α, 100 IU/ml IFN-γ or a combination of both for 16 h before infection with HSV-2 MS. After virus adsorption cells were re-fed with fresh cytokines which remained until 24 h p.i.. Different CDM treatments were performed: (a) Cells were treated with CDM 2h prior to interferon induction and then removed; (b) CDM was added simultaneously with IFN and remained only for 16 h before infection; (c) CDM was added after virus infection and remained until virus harvest; (d) CDM was added with IFN 16 h before infection, re-added after infection and remained to virus harvest. Average fold inhibition was calculated as (average viral titers in vehicle-treated/average viral titers in IFN-treated).

**Figure 4:**
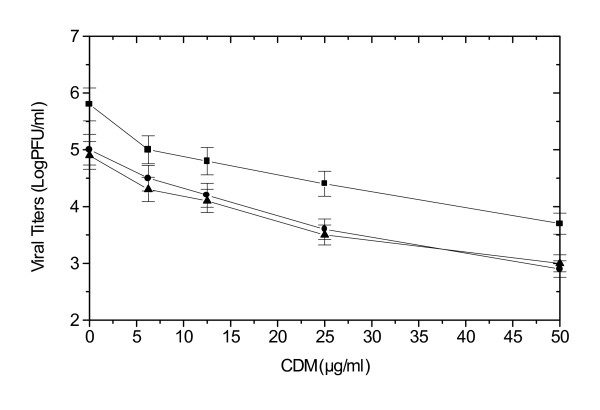
Additive effect of CDM and IFNs on HSV-2 inhibition. Several concentrations of CDM were added after HSV-2 MS infection in Vero cells treated 16 h before with vehicle, 100 IU/ml of IFN-α or 100 IU/ml of IFN-γ. Fresh IFN was re-added after infection too. Virus yields at 24 h p.i. were determined by plaque reduction assay. (■) CDM alone, (●) CDM + 100 IU/ml of IFN-α; (▲) CDM + 100 IU/ml of IFN-γ.

We also tested if the presence of CDM under different experimental conditions could affect the antiviral action of IFNs on HSV-2 MS replication. Virus yields at 24 h p.i. were determined in Vero cells treated with CDM as follows: *i*) for 2 h prior to interferon induction and then removed, *ii*) added simultaneously with IFN and remained only for 16 h before infection, *iii*) added after virus infection and remained to virus harvest, *iiii*) added with IFN 16 h before infection, re-added after infection and remained to virus harvest. The results obtained following protocol *i*) are depicted in Figure 3a. The antiviral effect due to interferon alone or in combination is 2-fold increased in cells pretreated with CDM, as an indication that CDM treatment did not interfere with the antiviral activity displayed by IFN alone or in combination. On the contrary, we can speculate that the antiviral effect of CDM alone contributed in an additive manner to the overall antiviral activity. When CDM was present during the 16 h IFN antiviral induction period, the interpretation of the results are rather complicated because of the antiviral effect of CDM *per se *which reduced HSV-2 replication by 20-fold (Figure 3b). Likewise, in the experiments described in Figure 3a, there is an additive effect of CDM when cells were treated with both IFN-α or IFN-γ or their combination (Figure 3b). When CDM was added after virus infection and remained until 24 h p.i. or it was also present during the induction period, the antiviral effect of the compound was so high (100-fold and 20.000-fold reduction virus yield respectively) that masked its interaction with IFNs (Figure 3c and 3d). For that reason, we repeated the experiment shown in Figure 3c with lower concentrations of CDM. Virus yields at 24 h p.i. in cultures treated with 6.12, 12.25, 25 and 50 μg/ml of CDM, or CDM plus 100 IU/ml of IFN-α or CDM plus 100 IU/ml of IFN-γ were determined. As can be seen in Figure 4, CDM alone inhibited virus replication in a dose dependent manner. The combination of each IFN with CDM increased in 1 log the antiviral effect but without altering the kinetics of the inhibition observed.

All these results suggest that the presence of CDM did not interfere with the anti-HSV-2 inhibition of IFN-α or IFN-γ alone or in combination.

## Discussion

Binding of IFN-α/β or IFN-γ to their specific cell receptors modified the transcriptional and translational environments such that an antiviral state is induced [26]. However, nearly all the animal viruses evolved mechanisms to antagonize the effect to IFN-induced antiviral state [27]. In the case of HSV-1 it was reported that the resistance to IFNα/β is an active process that is dependent on the expression of at least two viral proteins the immediate early protein ICP0 [28,29] and ICP34.5 [30,31]. Expression of ICP0 also plays a role in resistance of HSV-1 to IFN-γ[32]. Thus explaining why IFNα/β or IFN-γ alone are weak inhibitors of HSV-1 wild type virus replication. Strikingly, recent studies showed that co-activation of IFNα/β and IFN-γ receptors renders cells highly resistant to HSV-1 replication [20]. This finding is turning to be a general phenomenon since a synergistic antiviral effect of the combination of IFN-α and IFN- γ was also demonstrated for other members of *Herpesviridae *[20,23,33] and a variety of RNA viruses like SARS [34], Lassa virus [35] and HCV [25].

Considering this background the current investigation was undertaken to determine if IFN combination inhibited in a synergistic manner HSV-2 replication in Vero cells, and if so to determine if the presence of the plant derived antiviral compound CDM could affect the antiviral state induced by IFN-α or IFN-γ or their combination.

As expected, the results shown in Table 1, Figure 1a and Figure 2b, clearly demonstrated that HSV-2 plaque formation, viral replication and the onset of viral CPE in Vero cells are synergistically inhibited by interferon combination. Whereas, individual cytokines were weak inducers of the antiviral response, independently of the concentration tried was 100 or 200 IU (Table 1). Importantly, there is no evidence that the two cytokines, individually or collectively, were harmful to uninfected or HSV-2 infected cells at the concentration used (Figure 2a and 2b). To the contrary, the cells remained viable after cytokine exposure for 48 h after the induction period as assessed by MTT exclusion colorimetric method and remained able to proliferate (Figure 2a) indicating that virus inhibition was the consequence of a blockade in virus replication and no to host cells death. The validity of these findings was provided by the inclusion of HSV-1 KOS and HSV-1 F strains in the experiments as controls. Results depicted in Table 1, Figures 1c and 1g (KOS), 1d and 1 h (F) showed a synergistic effect of IFN combination on HSV-1 virus replication confirming previous studies [36,20].

The antiviral effect of CDM in infected cells is due to alterations in cellular processes. Working with VSV as a model system it was demonstrated that CDM exerted its antiviral action on the endocytic and exocytic pathway of VSV by pre- or post-treatment [37]. This effect is a consequence that CDM induced cytoplasmic alkalinization of intracellular endosomes. The refractory state to virus infection reached a maximum after 2 h of pre-treatment and is fully maintained up to 12 h later, however even at 24 h the cells still remain partially resistant to virus infection. Since acidification of vacuolar compartments plays an important role in a variety of cellular processes we wondered where the presence of CDM could inhibit the antiviral state induced by interferon treatment.

The results presented in Figure 2a showed that a concentration of 50 μg/ml of CDM alone or in combination with interferon did not affect uninfected cell viability. Moreover, the presence of CDM, either alone or in combination, for 48 h (Figures 2b and 2c) contributes to protect cells from virus CPE. So, the results obtained were not perturbed by compounds cytopathicity.

To test the antiviral action of IFN we used a standard procedure comprising a period of 16 h cell treatment (induction of the antiviral state) previous to virus infection. IFN was then removed during virus adsorption and after that re-added up to virus harvest. The presence of CDM for 2 h before induction, during the 16 h induction period, only for 24 h after infection or during the complete IFN treatment period did not affect IFN action. On the contrary, in the presence of CDM, an enhanced effect of the antiviral action of each interferon alone or in combination (see Figure 3) was observed indicating that alteration of cellular processes occurring in cells after exposure to CDM [37] did not interfere with the establishment of a refractory stage to HSV-2 infection in cells treated with IFN. It is remarkable that the sensitivity of HSV-2 to CDM increased steadily in accordance with time of cell treatment. Thus, after 2 h pretreatment with CDM virus titer was 2 fold reduced (Figure 3a) but if pretreatment was extended to 16 h, virus reduction increased 10 times (Figure 3b). In accordance with previous results obtained with meliacine [5], addition of CDM to cells after HSV-2 infection caused a strong inhibition of virus titer in the order of 100 fold (Figure 3c). Unexpectedly, when CDM was present for 16 h before infection and for 24 h afterwards, virus yield was reduced 20.000 times (Figure 3d). This high antiviral activity of CDM *per se *complicated the interpretation of the results obtained in the presence of IFN (Figure 3d), which in comparison reduced the amount of virus even in combination less than 2 logs. To overcome this inconvenience, we performed an experiment with lower concentrations of CDM added after virus infection and lasted to virus harvest, in combination with 100 IU/ml of IFN-α or IFN-γ. A clear additive effect of CDM with IFN-α or CDM plus IFN-γ at all concentrations tested was observed (Figure 4).

Thus, the results reported here indicated that the presence of CDM did not alter the biological activity of IFN-α or IFN-γ or their combination. As a result we can envision that the administration of CDM *in vivo *could not affect the production of IFNs, which are so important mediators on the innate resistance to HSV-2 infection [19].

## Conclusion

We have shown here that IFN-α, together with IFN-γ synergistically inhibits the replication of HSV-2 *in vitro *like it was also demonstrated for HSV-1 and others DNA and RNA viruses. On the other hand, we have shown too that CDM display a high antiviral activity against HSV-2 without interfering with the biological activity of IFN. We hypothesize that *in vivo *administration of CDM could not affect the antiviral activity of interferons to inhibit HSV-2 replication in the mice genital tract.

## Methods

### Cells and viruses

Vero cells were grown in Eagle's minimum essential medium supplemented with 5% inactivated calf serum (MEM 5%) and gentamycin (50 μg/ml) at 37°C in 5% CO_2 _and maintained after monolayer formation in MEM supplemented with 1.5% inactivated calf serum (MEM 1.5%).

Wild type HSV-1 KOS strain was a gift from Dr Erik De Clercq (Rega Institute, Leuven, Belgium). HSV-1 strain F and HSV-2 strains MS and G were obtained from American Type Culture Collection and propagated in Vero cells

### Interferons

Recombinant human Interferon.alpha-2 b (Sidus, Buenos Aires, Argentina) and Interferon-gamma 1B (Boehringer Ingelheim, Ingelheim, Germany) were added to cultures 16 h before infection, replaced after HSV infection and maintained until supernatant harvest. In all experiments 100 IU/ml of each interferon were used unless stated otherwise.

### Antiviral compound

CDM (1-cinnamoyl-3,11-dihydroxymeliacarpin) was purified in our laboratory from the leaves of *M.azedarach *L, as described by Alché *et al*. [22]. Solubilized in MEM 1.5% to a final concentration of 1 mg/ml and stored at -70°C. CDM was used at a concentration of 50 μg/ml (75 μM).

### Viral plaque reduction assays

For plaque reduction assay Vero cells were seeded in 24-well plates at a density of 10^5 ^cells per well, and 12 h later 100 IU/ml of IFN-α or IFN-γ or both IFN-α and IFN-γ (100 IU/ml of each) were added to the culture medium. Vero cells were inoculated with HSV-2 (strains MS and G) or HSV-1 (strains KOS and F) 16 h later, and after adsorption the medium was replaced with complete MEM containing 1.5% methylcellulose and the same IFNs used in the pretreatment. Plaques were counted two days later.

### Viral replication assays

For virus replication assays, Vero cells were seeded in 24-well plates at a density of 10^5 ^cells per well, and 12 h later cultures were treated with vehicle, 100 IU/ml of IFN-α and/or 100 IU/ml of IFN-γ. After 16 h of IFN treatment, cell monolayers were inoculated with HSV-2 (strains MS and G) or HSV-1 (strains KOS and F) at a multiplicity of infection (MOI) of 1 PFU per cell. After 1 h adsorption, the inoculum was removed and fresh IFN-containing culture medium was returned to each well. Twenty-four, 48 or 72 h p.i., titers of infectious virus in cell supernatants were determined by serial dilution plaque assay on Vero cells.

To test the effect of CDM on IFN antiviral action we performed different experimental schedule. Vero cells were treated with CDM as follows: *i) *for 2 h prior to interferon induction and then removed, *ii*) added simultaneously with IFN and remained only for 16 h before infection, *iii*) added after virus infection and remained to virus harvested, *iiii*) added with IFN 16 h before infection, re-added after infection and remained to virus harvested.

### Enumeration of viable cells

Vero cells were established at a density of 2 × 10^4 ^cells/well in 96-well plates and 12 h later fresh culture medium or medium containing 50 μg/ml of CDM or 100 IU/ml of IFN-α or IFN-γ or both IFN-α and IFN-γ (100 IU/ml of each) alone or in combination with 50 μg/ml of CDM were added to the culture medium. Vero cells were inoculated with HSV-2 MS and HSV-1 KOS 16 h later, and after virus adsorption the medium was replaced with fresh culture medium containing the same IFN concentrations used in the pretreatment. At 0, 12, 24, 36 and 48 h p.i. cell morphology was observed by light microscope and cell viability was determined as described previously [38] using the cleavage of tetrazolium salt MTT [3-(4,5-dimethylthiazol-2-yl)-2,5-diphenyl tetrazolium bromide] (Sigma) by the mitochondrial enzyme succinate dehydrogenase to give a blue product (formazan). The absorbance of each well was measured on an Eurogenetics MPR-A 4i microplate reader, using a test wavelength of 570 nm and a reference wavelength of 630 nm. The number of surviving cells was determined by interpolation in a standard calibration curve correlating optical density values and number of viable cells determined by counting with a haemocytometer.

### Statistics

Data are presented as the means ± standard error of the means (sem). Data from IFN-treated groups were compared to vehicle-treated groups and significant differences were determined by one-way analysis of variance (ANOVA) followed by Turkey's post hoc *t *test (GraphPad Prism^© ^Home, San Diego, CA).

## Competing interests

The author(s) declare that they have no competing interests.

## Authors' contributions

EP participated in the experimental design, performed all experiments and drafted the manuscript. CEC conceived and design of the study, and drafted the manuscript. Both authors read and approved the final manuscript.

## References

[B1] Waltz MA, Price RW, Hayashi K, Katz BJ, Notkins AL (1977). Effect of immunization on acute and latent infections of vagino-uterine tissue with herpes simplex virus type 1 and 2. J Infect Dis.

[B2] Richards JT, Kern ER, Overall JC, Glasgow LA (1981). Differences in neurovirulence among isolates of herpes simplex virus types 1 and 2 in mice using four routs of infection. J Infect Dis.

[B3] Smee DF, Martin JC, Verheyden JPH, Matthews TR (1983). Anti-herpesvirus activity of the acyclic nuclioside 9-(1,3-dihydroxy-2-propoxymethyl) guanine. Antimicrobial Agents and Chemotherapy.

[B4] Whaley KJ, Barratt RA, Zeitlin L, Hoen TE, Cone RA (1993). Nonoxynol-9-protects mice against vaginal transmission of genital herpes infections. J Infect Dis.

[B5] Andrei G, Couto AS, de Lederkremer RM, Coto CE (1994). Purification and partial characterization of an antiviral active peptide from Melia azedarach L. Antivir Chem Chemother.

[B6] Barquero AA, Alché LE, Coto CE (1997). Antiviral activity of meliacine on the replication of a thymidine kinase-deficient mutant of herpes simplex virus type 1 alone and in combination with acyclovir. Int J Antimicrob Agents.

[B7] Alché LE, Barquero AA, San Juan NA, Coto CE (2002). An antiviral principle present in a purified fraction from Melia azedarach L leaves aqous extracts restreing herpes simplex virus type 1 propagation. Phytother Res.

[B8] Alché LE, Berra A, Beloso MJ, Coto CE (2000). Treatment with meliacine, a plant derived antiviral, prevents the development of herpetic stromal keratitis in mice. J Med Virol.

[B9] Pifarré MP, Berra A, Coto CE, Alché LE (2002). Therapeutic action of meliacine, a plant-derived antiviral, on HSV-induced ocular disease in mice. Exp Eye Res.

[B10] Petrera E, Coto CE (2004). Efectos de la administración in vivo del antiviral meliacina sobre el curso de la infección herpética genital murina [abstract]. Medicina.

[B11] Courrèges MC, Benencia F, Coto CE, Massouh EJ, Coulombié FC (1994). In vitro antiphagocytic effect of Melia azedarach leaf extracts on mouse peritoneal exudate cells. J Ethnopharmacol.

[B12] Courrèges MC, Benencia F, Coulombié FC, Coto CE (1998). In vitro and in vivo activities of Melia azedarach L. aqueous leaf extracts on murine lymphocytes. Phytomedicine.

[B13] Petrera E, Coto CE (2003). Effect of meliacine, a plant derived antiviral, on tumor necrosis factor alpha. Fitoterapia.

[B14] Ellerman-Eriksen S (1993). Autocrine secretion of interferon-alpha/beta and tumor necrosis factor-alpha synergistically activates macrophages after infection with herpes simples virus type 2. J Gen Virol.

[B15] Bohem U, Klamp T, Groot M, Howard JC (1997). Cellular response to interferon-γ. Annu Rev Immunol.

[B16] Paludan SR, Ellermann-Eriksen S, Mogensen SC (1998). NF-kappaB activation is responsible for the synergistic effect of herpes simplex virus type 2 infection on interferon-gamma-induced nitric oxide production. J Gen Virol.

[B17] Malmgaard L, Paludan SR, Mogensen SC, Ellermann-Eriksen S (2000). Herpes simplex virus type 2 induces interleukin-12 in macrophages through a mechanism involving NF-κB. J Gen Virol.

[B18] Vollstedt S, Arnold S, Schwerdel C, Franchini M, Alber Gottfried, Di Santo JP, Ackermann M, Suter M (2004). Interplay between alpha/beta and gamma interferons with B, T and natural killer cells in the defense against herpes simplex virus type 1. J of Virol.

[B19] Malmgaard L, Paludan SR (2003). Interferon (IFN)-α/β, interleukin (IL)-12 and IL-18 coordinately induce production of IFN-γ during infection with herpes simplex virus type 2. J Gen Virol.

[B20] Sainz B, Halford W (2002). Alpha/beta interferon and gamma interferon synergize to inhibit the replication of herpes simplex virus type 1. J of Virol.

[B21] Mikloska Z, Cunningham AL (2001). Alpha and gamma interferons inhibit herpes simplez virus type I infection and spread in epidermal cells after axonal tranmission. J Virol.

[B22] Alché LE, Ferek GA, Meo M, Coto CE, Meier MS (2003). An antiviral meliacarpin from leaves of Melia azedarach L. Z Naturforsch.

[B23] Pierce AT, De Salvo J, Foster TP, Kosinski A, Séller SK, Halford WP (2005). Beta interferon and gamma interferon synergize to block DNA and virion synthesis in herpes simplex virus-infected cells. J Gen Virol.

[B24] Desloges N, Rahaus M, Wolff MH (2005). Role of protein kinase PKR in the inhibition of varicella-zoster virus replication by beta interferon and gamma interferon. J Gen Virol.

[B25] Sainz B, LaMarca HL, Garry RF, Morris C (2005). Synergistic inhibition of human cytomegalovirus replication by interferon-alpha/beta and interferon-gamma. Virol J.

[B26] Samuel CE (2001). Antiviral action of interferons. Clinical Microbiology Reviews.

[B27] Tan SL, Katze MG (2000). HSV.com: maneuvering the internetworks of viral neuropathogenesis and evasion of the host defense. Proc Natl Acad Sci U S.

[B28] Leib DA, Harrison TE, Laslo KM, Machalek MA, Moorman NJ, Virgin HW (1999). Interferons regulate the phenotype of wild-type and mutant herpes simplex virus in vivo. J Exp Medicine.

[B29] Mossman KL, Sherburne R, Lavery C, Duncan J, Smiley JR (2000). Evidence that herpes simplex virus VP16 is required for viral egress downstream of the initial envelopment event. J Virol.

[B30] Cassady KA, Gross M, Roizman B (1998). The second-site mutation in the herpes simplex virus recombinants lacking the gamma134.5 genes precludes shutoff of protein synthesis by blocking the phosphorylation of eIF-2alpha. J Virol.

[B31] Leib DA, Machalek MA, Williams BR, Silverman RH, Virgin HW (2000). Specific phenotypic restoration of an attenuated virus by knockout of a host resistance gene. Proc Natl Acad Sci U S A.

[B32] Härle P, Sainz B, Carr DJ, Halford WP (2002). The immediate-early protein, ICP0, is essential for the resistance of herpes simplex virus to interferon alpha/beta. Virol.

[B33] Halford WP, Halford KJ, Pierce AT (2005). Mathematical analysis demonstrates that interferons-beta and -gamma interact in a multiplicative manner to disrupt herpes simplex virus replication. J Theor Biol.

[B34] Sainz B, Mossel EC, Peters CJ, Garry RF (2004). Interferon-beta and interferon-gamma synergistically inhibit the replication of severe acute respiratory syndrome-associated coronavirus (SARS-CoV). Virol.

[B35] Asper M, Sternsdorf T, Hass M, Drosten C, Rhode A, Schmitz H, Günther S (2004). Inhibition of different Lassa virus strains by alpha and gamma interferons and comparison with a less pathogenic arenavirus. J Virol.

[B36] Balish MJ, Abrams ME, Pumfery AM, Brandt CR (1992). Enhanced inhibition of herpes simplex virus type 1 growth in human corneal fibroblasts by combinations of interferon-alpha and -gamma. J Infect Dis.

[B37] Barquero AA, Alché LE, Coto CE (2004). Block of vesicular stomatitis virus endocytic and exocytic pathways by 1-cinnamoyl-3,11-dihydroxymeliacarpin, a tetranortriterpenoid of natural origin. J Gen Virol.

[B38] Petrera E, Joselevich M, Ghini A, Burton G, Coto CE (2003). Antiherpes virus activities of new 6–19 carbon-bridged steroids and some synthetic precursors. Antivir Chem Chemother.

